# Interleukin-13 +1923C/T Polymorphism Is Associated with Asthma Risk: A Meta-Analysis

**DOI:** 10.1155/2013/394316

**Published:** 2013-06-11

**Authors:** Yongan Liu, Tao Liu, Wei Nie, Guoxiang Lai, Qingyu Xiu

**Affiliations:** ^1^Department of Intensive Care Medicine, No. 411 Hospital of PLA, Shanghai 200080, China; ^2^Department of Respiratory Disease, Shanghai Changzheng Hospital, Second Military Medical University, 415 Fengyang Road, Shanghai 200003, China; ^3^Department of Respiratory Disease, Fuzhou Military General Hospital, Fuzhou, Fujian 350025, China

## Abstract

There are controversies on the association between *interleukin-13* (*IL-13*) +1923C/T polymorphism (rs1295686) and the risk of asthma. We performed this study to assess the association by the method of meta-analysis. A systematic search current to October 16, 2012, was conducted using PubMed, EMBASE, and China National Knowledge Infrastructure (CNKI) and identified ten studies comprising 13698 cases and 38209 controls. The pooled odds ratios (ORs) with 95% confidence intervals (CIs) were calculated. There was a significant association between *IL-13* +1923C/T polymorphism and asthma risk in codominant model. When stratified by ethnicity, *IL-13* +1923C/T polymorphism remained significantly associated with higher asthma risk in Asians and Caucasians. In the subgroup analysis by study quality, a significantly increased asthma risk was observed in high quality studies. Sensitivity analysis and cumulative analysis further strengthened the validity of the results. No publication bias was found in this meta-analysis. In conclusion, results from this meta-analysis suggested that *IL-13* +1923C/T polymorphism was a risk factor of asthma.

## 1. Introduction

The prevalence of asthma has rapidly increased over the last few decades to epidemic proportions and there are approximately 300 million people worldwide [1]. The risk of developing asthma tends to run in families, and heritability of asthma has been estimated as 60% [2]. Thus, host genetic susceptibility may play a crucial role in the pathogenesis of asthma. Until now, many studies have focused on this field, and the *interleukin-13* (*IL-13*) gene has been extensively studied.

*IL-13* has been demonstrated to be the central mediator of allergic asthma [3, 4]. In asthmatic patients, Huang et al. [5] found that the expression of *IL-13* was increased in the allergen-challenged bronchoalveolar lavage (BAL). Similarly, a significant increase in the expression of *IL-13* mRNA in BAL cells enriched for alveolar macrophages of the asthmatic patients was observed [6]. Recently, Saha and coworkers suggested that *IL-13* overexpression in sputum and bronchial biopsy specimens was a feature of severe asthma [7]. Furthermore, *IL-13* expression was related to asthma control and the intensity of eosinophilic inflammation [7]. Collectively, these results indicated that *IL-13* might have an important role in the pathophysiology of asthma.

 The *IL-13 *gene is located on chromosome 5q31. Two single nucleotide polymorphisms (SNPs) of *IL-13* have been investigated in relation to asthma. One is located in the promoter region at position −1112, the other is a G>A transition at nucleotide +2044 in the coding region of exon 4 [8]. Two meta-analyses assessing associations between these polymorphisms and asthma risk have be published [9, 10]. However, the role of *IL-13* +1923C/T polymorphism (rs1295686) on risk of asthma was still unknown. Previous studies indicated that *IL-13* +1923C/T polymorphism was not associated with susceptibility to asthma [11, 12]. By contrast, recent studies suggested that this polymorphism played a critical role in the development of asthma [13, 14]. Therefore, we conducted a meta-analysis of all available case-control studies to evaluate the association of *IL-13* +1923C/T polymorphism with asthma risk.

## 2. Methods

### 2.1. Publication Search

The electronic databases Pubmed, EMBASE, and China National Knowledge Infrastructure (CNKI) were searched using the following terms: (asthma or asthmatic) and (interleukin-13 or interleukin 13 or *IL-13* or IL13) and (polymorphism or mutation or variant). Last search was updated in October 16, 2012. No publication date or language restrictions was imposed. We reviewed the bibliographies of all selection articles to identify additional relevant studies.

### 2.2. Inclusion and Exclusion Criteria

All selected studies complied with the following three criteria: (1) evaluation of the +1923C/T polymorphism in *IL-13* gene and asthma risk; (2) using a case-control design; (3) sufficient data for estimating for estimating an odds ratio (OR) and 95% confidence interval (CI).

Studies were excluded if one of the following existed: (1) not relevant to *IL-13* +1923C/T polymorphism or asthma risk, (2) nonclinical study, (3) genotype frequencies or number not reported, and (4) reviews and abstracts. For the overlapping studies, the largest or most recent publication was selected.

### 2.3. Data Extraction

Two investigators (Yongan Liu and Tao Liu) independently extracted the following data from each included study: the first author's name, year of publication, original country, ethnicity, age, atopic status, sample size, genotyping method, and genotype number in cases and controls. We verified accuracy of data by comparing collection forms from each investigator. Agreement was reached after discussion for conflicting data or a third author (Wei Nie) would assess these articles.

### 2.4. Quality Assessment

The quality of included studies was assessed independently by two investigators (Wei Nie and Guoxiang Lai) using a quality scoring system. Table S1 in Supplementary Material available online at http://dx.doi.org/10.1155/2013/394316 shows the criteria for quality appraisal. The quality scoring system was based on traditional epidemiological considerations and asthma genetic issues [15]. The criteria covered the representativeness of cases and controls, the ascertainment of cases and controls, genotyping examination, Hardy-Weinberg equilibrium (HWE), association assessment, and response rate. Scores were ranged from 0 (worst) to 15 (best). We defined the study with scores >6 was high quality, and the study with scores ≤6 was low quality study.

### 2.5. Statistical Analysis

When the data from at least three studies were available, a meta-analysis was performed. ORs with 95% CIs were computed to assess the strength of the correlation between the *IL-13* +1923C/T polymorphism and asthma risk. The statistical significance of OR was analyzed by *Z* test. OR1, OR2, and OR3 regarding *IL-13* +1923C/T polymorphism were calculated for genotypes TT versus CC, TC versus CC, and TT versus TC, respectively. These pairwise differences were used to indicate the most appropriate genetic model as follows: if OR1 = OR3 *≠* 1 and OR2 = 1, then a recessive model was suggested; if OR1 = OR2 *≠* 1 and OR3 = 1, then a dominant model was suggested; if OR2 = 1/OR3 *≠* 1 and OR1 = 1, then a complete overdominant model was suggested; if OR1 > OR2 > 1 and OR1 > OR3 > 1 (or OR1 < OR2 < 1 and OR1 < OR3 < 1), then a codominant model was suggested [16–18]. Once the best genetic model was identified, this model was used to collapse the three genotypes into two groups (except in the case of a codominant model) and to pool the results again.

Departure from HWE in controls was tested by the Chi-square test. The Q statistic was used to test for heterogeneity between the studies, which is considered to be significant for *P* < 0.10. *I* ^2^ statistics were also used to investigate the degree of heterogeneity among studies. Subgroup analyses were performed by ethnicity and study quality. We defined the subjects from East Asia were Asians. We also defined people from Europe, Northern America, Oceania, North Africa, the Horn of Africa, Western Asia, and Central Asia were Caucasians. Sensitivity analysis was conducted by omitting each study in turn. We also performed cumulative meta-analysis to evaluate the trend of summary ORs (95% CIs) by the year of publication. Egger's test [19] was used to evaluate publication bias.

 All statistical tests were performed by using the RevMan 5.1 software (Nordic Cochrane Center, Copenhagen, Denmark) and STATA 11.0 software (Stata Corporation, College Station, TX). A *P* value < 0.05 was considered statistically significant.

## 3. Results

### 3.1. Study Characteristics

**Figure 1:**
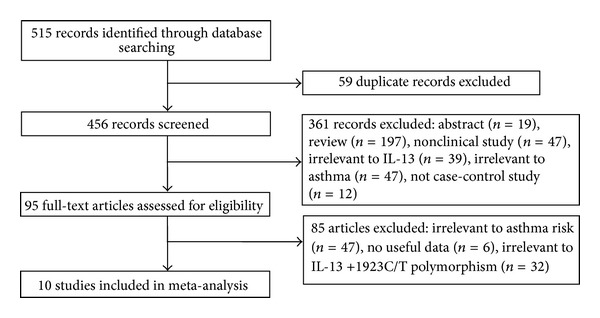
Flow of study identification, inclusion, and exclusion.

Figure 1 outlines our study selection process. A total of 515 articles were identified after searching and screening. After careful review, ten eligible case-control studies on the relationship between *IL-13* +1923C/T polymorphism and asthma risk were included in this meta-analysis [11–14, 20–25]. The studies conducted by Donfack et al. [12] and Yoon et al. [25] reported two cohorts, and each cohort was considered as a case-control study. Finally, 13698 asthmatic cases and 38209 controls were included in this study. The characteristics of each study are exhibited in Table 1. There were four studies of Asians [20, 23–25], five studies of Caucasians [11–14, 22], and two studies of African Caucasian [12, 21]. Three studies used adult population [14, 20, 25], two studies used child population [23, 24], and four studies included both adults and children [11, 13, 21, 22]. Only one study was performed in atopic patients [11]. Two studies was defined low quality [20, 23], while seven studies were high quality [11–14, 21, 22, 24, 25], suggesting acceptable methodology quality. Genotype numbers and HWE examination results are listed in Table 2.

### 3.2. Quantitative Data Synthesis

**Figure 2:**
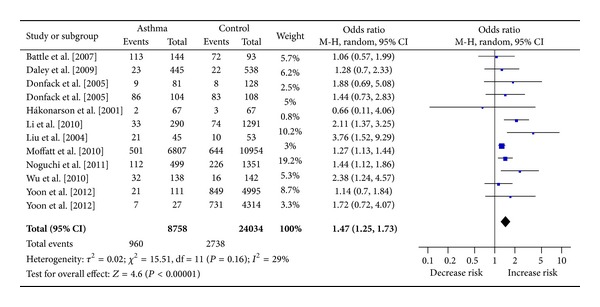
Meta-analysis for the association between asthma risk and the *IL-13* +1923C/T polymorphism (TT versus CC).

**Figure 3:**
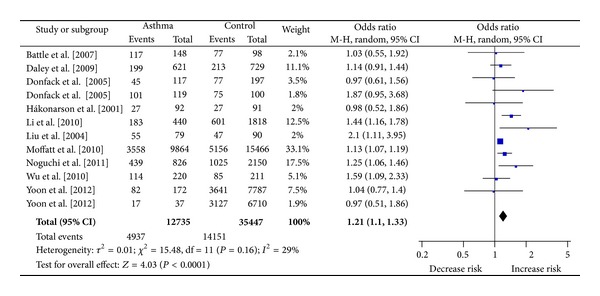
Meta-analysis for the association between asthma risk and the *IL-13* +1923C/T polymorphism (TC versus CC).

The estimated OR1, OR2 and OR3 were 1.47, 1.21, and 1.14, respectively (Table 3). These estimates suggested a codominant genetic model. Thus, there was no need to to collapse the three genotypes into two groups and to pool the results again. As shown in Figure 2, a significant increased asthma risk was observed for TT versus CC (OR = 1.47, 95% CI 1.25–1.73, *P* < 0.00001, *I* ^2^ = 29.0%). As for TC versus CC (Figure 3), the result was also significant (OR = 1.21, 95% CI 1.10–1.33, *P* < 0.0001, *I* ^2^ = 29.0%).

### 3.3. Subgroup Analysis

In the subgroup analysis by ethnicity, significant associations were found among Asians (OR = 1.67, 95% CI 1.20–2.34, *P* = 0.003, *I* ^2^ = 45.0%) and among Caucasians (OR = 1.45, 95% CI 1.11–1.88, *P* = 0.006, *I* ^2^ = 33.0%) for TT versus CC. Similarly, significant associations were also observed among Asians (OR = 1.27, 95% CI 1.05–1.55, *P* = 0.01, *I* ^2^ = 35.0%) and among Caucasians (OR = 1.17, 95% CI 1.05–1.30, *P* = 0.004, *I* ^2^ = 27.0%) for TC versus CC. In the stratified analysis by study quality, statistically significant associations were found in the studies with high quality for TT versus CC (OR = 1.33, 95%CI 1.21–1.47, *P* < 0.00001, *I* ^2^ = 0%) and for TC versus CC (OR = 1.15, 95% CI 1.09–1.22, *P* < 0.00001, *I* ^2^ = 3.0%), respectively. Summary of meta-analysis results is presented in Table 3.

### 3.4. Sensitivity Analysis and Cumulative Meta-Analysis

**Figure 4:**
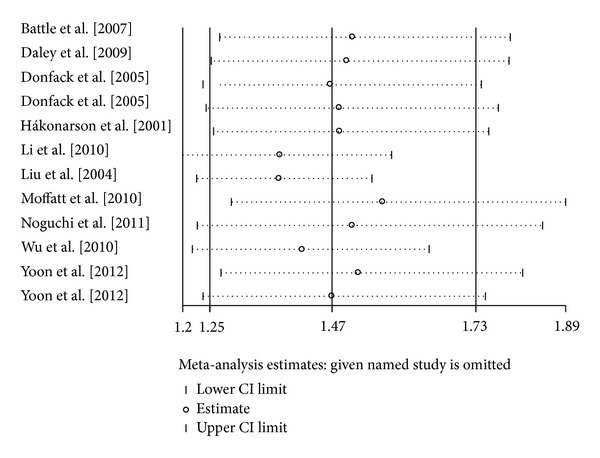
Sensitivity analysis for the *IL-13* +1923C/T polymorphism with asthma risk (TT versus CC).

**Figure 5:**
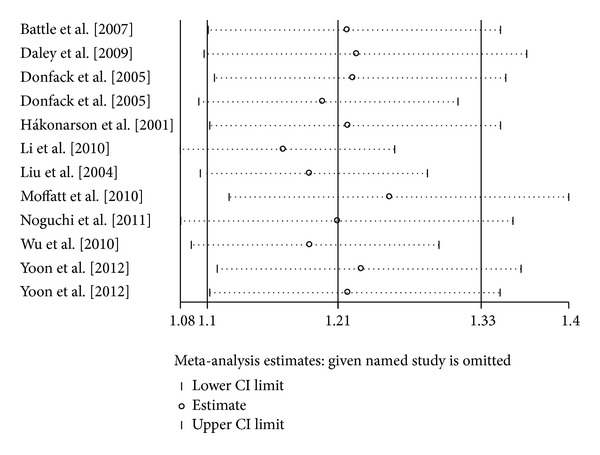
Sensitivity analysis for the *IL-13* +1923C/T polymorphism with asthma risk (TC versus CC).

**Figure 6:**
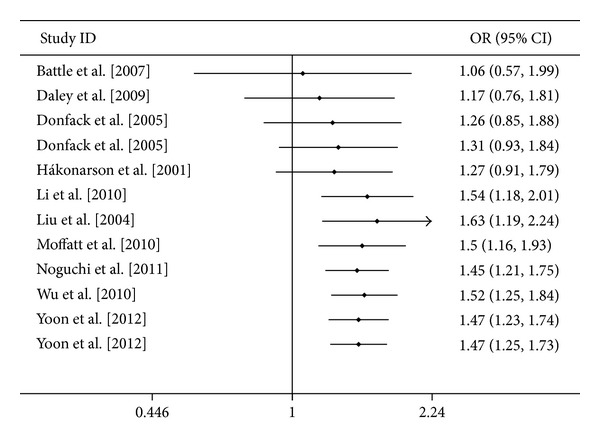
Cumulative meta-analysis of associations between *IL-13* +1923C/T polymorphism and asthma risk (TT versus CC).

**Figure 7:**
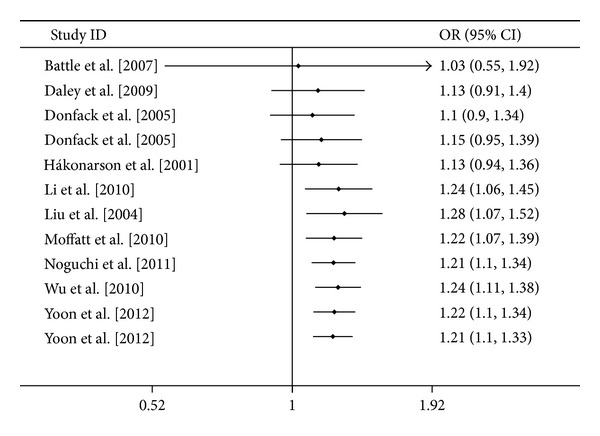
Cumulative meta-analysis of associations between *IL-13* +1923C/T polymorphism and asthma risk (TC versus CC).

Sensitivity analyses were conducted repeatedly when each particular study was omitted. As shown in Figure 4, the results were not materially altered, with pooled ORs ranging from 1.37 to 1.56 for TT versus CC. Similarly, there was little modification of the estimates after exclusion of individual study, with pooled ORs ranging from 1.19 to 1.25 for TC versus CC (Figure 5). We also performed cumulative meta-analyses by pooling data again, and each time a study was added. The results showed that the pooled ORs tended to be stable (Figures 6 and 7).

### 3.5. Publication Bias

No publication bias was detected. *P* values were 0.155 and 0.216 in Egger's test, separately.

## 4. Discussion

Strong experimental evidence demonstrated that *IL-13* could direct many of the important features of airway inflammation and remodeling in asthma. Introduction of exogenous *IL-13* into murine airways resulted in lymphocytic and eosinophilic inflammation, airway remodeling, and airway hyperresponsiveness (AHR) [3, 4]. By contrast, knocking out *IL-13* in mice prevented the development of AHR after allergen exposure [26]. Furthermore, using a mouse model of chronic asthma, Yang et al. [27] found that *IL-13* antibody significantly suppressed AHR, eosinophil infiltration, proinflammatory cytokine/chemokine production, serum IgE, and airway remodeling. In addition, Corren and colleagues demonstrated that lebrikizumab (a monoclonal antibody to *IL-13*) treatment was associated with improved lung function in patients with asthma. These results strongly suggested that *IL-13* was a major effector of asthma. Recently, a genome-wide association study (GWAS) conducted by Moffatt et al. [13] found that *IL-13* +1923C/T polymorphism was associated with asthma risk and the total serum IgE concentration. More recently, another GWAS performed by Granada et al. [28] confirmed that *IL-13* +1923C/T polymorphism was a risk factor for IgE dysregulation. Specifically, Maier and colleagues indicated that the *IL-13* +1923T allele was associated with higher IgE level than the +1923C allele [29]. These results indicated the critical role of *IL-13 *+1923C/T polymorphism in the regulation of IgE level. IgE-dependent mechanisms play an important role in the development and maintenance of airway inflammation in asthma. It is biologically plausible that the *IL-13* +1923C/T polymorphism which can affect IgE level could influence the susceptibility to asthma. However, it is currently unclear whether the *IL-13 *+1923C/T polymorphism causes overexpression or enhances function of *IL-13*. Therefore, the functional studies of this polymorphism are required. A number of studies have investigated the association between *IL-13 *+1923C/T polymorphism and asthma risk, but the results were controversial and underpowered. Meta-analysis offers a powerful means of overcoming problems such as small sample sizes and the inadequate statistical powers of genetic studies on complex disorders. Therefore, we performed this meta-analysis to assess the association between *IL-13 *+1923C/T polymorphism and asthma risk.

 This meta-analysis, including a total of 13698 cases and 38209 controls, examined the association between the *IL-13* +1923C/T polymorphism and asthma risk. We found that TT genotype and TC genotype were significantly associated with higher asthma risk, respectively. Compared with CC genotype, the carriers of the TT genotype had 47% increased asthma risk. As for the individuals carrying with TC genotype, they had 21% elevated asthma risk. These findings suggested that the carriers with the T allele of the *IL-13* +1923C/T polymorphism might be predisposed to asthma. In the subgroup analysis by race, significant associations were observed in Asians and Caucasians. There were only two studies in African Americans for this polymorphism [12, 21]. Therefore, subgroup analysis was not performed in African American subgroup. More studies with African American population are needed to evaluate the effect of *IL-13* +1923C/T polymorphism on asthma risk. In addition, we carried out subgroup analysis by study quality. *IL-13* +1923C/T polymorphism was still found to be associated with an elevated asthma risk in high quality studies. Because there were two studies with low quality, subgroup analysis was not performed.

 We carried out sensitivity analysis to assess the stability of this meta-analysis. Removal of each study did not alter the conclusion of increased asthma risk, suggesting the reliability of these results. Additionally, the cumulative meta-analyses were conducted. Results from the cumulative meta-analyses showed a trend of more obvious association between *IL-13* +1923C/T polymorphism and an increased risk of asthma as information accumulated by year. Therefore, we were convinced that the results of our meta-analysis were reliable.

 We noted that there was moderate heterogeneity in the overall comparisons in codominant genetic model. Thus, subgroup analysis was used to explore the sources of heterogeneity. We found that the *I* ^2^ values effectively reduced or disappeared when stratified by study quality. These results implicated that study quality may be the major source of the heterogeneity. Moreover, significant associations still existed in these high quality studies, which suggested that heterogeneity did not influence the results. In addition, it would be hard to interpret results, if significant publish bias was present. We assessed the publication biases by means of Egger's tests. However, we did not find obvious publication bias across the studies.

 Asthma is a complex inflammatory disease. It is unlikely that one SNP in one gene would be associated with asthma risk, without consideration of haplotype of the polymorphisms, environmental factors, or other polymorphic susceptible genes. For example, Sadeghnejad et al. [30] indicated that *IL-13* +1923C/T, +2044A/G, and +2525A/G polymorphisms were in strong linkage disequilibrium and were associated with raised cord serum IgE. Wu et al. [23] found the haplotype of *IL-13* +1923C/T polymorphism with the +2044A/G polymorphism affected susceptibility to asthma. In addition, there was a combined effect of *IL-13* gene polymorphisms and tobacco smoke on persistent childhood wheezing and asthma [31]. Furthermore, many other genes were related to asthma, such as *IL-4*, *IL-5*, and *PAI-1 *[8, 32]. However, haplotype analysis, gene-environment, and gene-gene interactions could not be addressed in this meta-analysis due to the lack of sufficient information.

 Some limitations must be pointed out. First, although no publication bias was found, selection bias could have occurred because some articles published in other languages (not English or Chinese) were not obtained and included in this meta-analysis. Second, several lines of evidence supported a central role for *IL-13* +1923C/T polymorphism in IgE dysregulation [27, 28]. Thus, it was possible that this polymorphism might associate with the risk of atopic asthma. However, we could not evaluate the association between *IL-13* +1923C/T polymorphism and atopic asthma because there was only one study performed in atopic asthmatic patients [11]. Third, there were only two studies of African American population in this meta-analysis and this population was not included in the subgroup analysis. Finally, different genotyping methods were used in the respective studies. This may be associated with different call rates.

To the best of our knowledge, this was the first meta-analysis to assess the relationship between the *IL-13* +1923C/T polymorphism and asthma susceptibility. Results from our study suggested that the *IL-13* +1923C/T polymorphism was significantly associated with the risk of asthma. Well-designed multicentre studies with more ethnicities are needed to validate our findings and study the potential effect modification by atopic status. Moreover, further investigations should also consider gene-gene and gene-environment interactions.

## Supplementary Material

This table shows the criteria for quality appraisal. The criteria covers the representativeness of cases and controls, the ascertainment of cases and controls, genotyping examination, Hardy-Weinberg equilibrium (HWE), association assessment, and response rate.

## Figures and Tables

**Table 1:** Characteristics of the case-control studies included in meta-analysis.

First author/reference	Year	Country	Ethnicity	Age group	Atopic status	Case (*n*)	Control (*n*)	Quality score	Genotyping method
Hákonarson [11]	2001	Iceland	Caucasian	Mixed	Atopic	94	94	10	PCR
Liu [20]	2004	China	Asian	Adult	NA	100	100	4	PCR-RFLP
Donfack 1 [12]	2005	USA	Caucasian	NA	Mixed	126	205	9	LAS
Donfack 2 [12]	2005	USA	African American	NA	Mixed	205	183	9	LAS
Battle [21]	2007	USA	African American	Mixed	NA	264	176	11	PCR-RFLP
Daley [22]	2009	Australia	Caucasian	Mixed	NA	644	751	9	Illumina Bead Array System
Wu [23]	2010	China	Asian	Children	NA	252	227	6	PCR-RFLP
Moffatt [13]	2010	Mixed	Caucasian	Mixed	NA	10365	16110	12	Illumina Human610 quad array
Li [14]	2010	USA	Caucasian	Adult	NA	473	1892	12	Illumina HumanCNV370 BeadChip
Noguchi [24]	2011	Japan	Asian	Children	Mixed	938	2376	12	Illumina HumanHap550v3/610-Quad Genotyping BeadChip
Yoon 1 [25]	2012	Korea	Asian	Adult	NA	193	8645	9	Affymetrix Genome-Wide Human SNP array 5.0
Yoon 2 [25]	2012	Korea	Asian	Adult	NA	44	7450	9	Affymetrix Genome-Wide Human SNP array 5.0

PCR: polymerase chain reaction; RFLP: restriction fragment length polymorphism; LAS: multiplex PCR and an immobilized linear array system; NA: not available.

**Table 2:** Distribution of *IL-13* +1923C/T genotype among patients and controls.

Study	Asthma			Control			HWE (*P* value)
	CC	CT	TT	CC	CT	TT	
Hákonarson et al. [11]	65	27	2	64	27	3	0.941
Liu et al. [20]	24	55	21	43	47	10	0.583
Donfack et al. 1 [12]	72	45	9	120	77	8	0.310
Donfack et al. 2 [12]	18	101	86	25	75	83	0.229
Battle et al. [21]	31	117	113	21	77	72	0.953
Daley et al. [22]	422	199	23	516	213	22	0.997
Wu et al. [23]	106	114	32	126	85	16	0.749
Moffatt et al. [13]	6306	3558	501	10310	5156	644	0.984
Li et al. [14]	257	183	33	1217	601	74	0.985
Noguchi et al. [24]	387	439	112	1125	1025	226	0.735
Yoon et al. 1 [25]	90	82	21	4146	3641	849	0.229
Yoon et al. 2 [25]	20	17	7	3583	3127	731	0.202

HWE: Hardy-Weinberg equilibrium.

**Table 3:** Determination of the genetic effect of *IL-13* +1923C/T on asthma and subgroup analyses.

Comparison	Study	Sample size		Number of studies	Test of association			Model	Heterogeneity		
		Case	Control		OR (95% CI)	*Z*	*P* value		*χ* ^2^	*P* value	*I* ^2^ (%)
TT versus CC	Overall	8758	24034	12	1.47 (1.25–1.73)	4.60	<0.00001	R	15.51	0.26	29.0
TC versus CC	Overall	12735	35447	12	1.21 (1.10–1.33)	4.03	<0.0001	R	15.48	0.16	29.0
TT versus TC	Overall	5897	16889	12	1.14 (1.04–1.25)	2.69	0.007	R	8.81	0.64	0.0
TT versus CC	Asian	820	10855	5	1.67 (1.20–2.34)	3.02	0.003	R	7.29	0.12	45.0
TT versus CC	Caucasian	7690	12978	5	1.45 (1.11–1.88)	2.75	0.006	R	5.97	0.20	33.0
TT versus CC	High quality	8575	23839	10	1.33 (1.21–1.47)	5.69	<0.00001	R	7.64	0.57	0.0
TC versus CC	Asian	1334	16948	5	1.27 (1.05–1.55)	2.46	0.01	R	6.15	0.19	35.0
TC versus CC	Caucasian	11134	18301	5	1.17 (1.05–1.30)	2.89	0.004	R	5.44	0.24	27.0
TC versus CC	High quality	12436	35146	10	1.15 (1.09–1.22)	5.13	<0.00001	R	9.28	0.41	3.0

R: random-effects model.
