# Supplementary material for: Interleukin-13 +1923C/T Polymorphism Is Associated with Asthma Risk: A Meta-Analysis
Source: Biomed Res Int. 2013 Jun 11;2013:394316. doi: 10.1155/2013/394316 (PMC3693103; doi:10.1155/2013/394316)
Supplement: Supplementary file 1 — This table shows the criteria for quality appraisal. The criteria covers the representativeness of cases and controls, the ascertainment of cases and controls, genotyping examination, Hardy-Weinberg equilibrium (HWE), association assessment, and response rate. [file 394316.f1.docx]

**Table S1**. Scale for quality assessment of molecular association studies of asthma

| Criteria | Score |
| --- | --- |
| **Representativeness of cases** |  |
| Consecutive/randomly selected from case | 2 |
| population with clearly defined sampling |  |
| frame |  |
| Consecutive/randomly selected from case | 1 |
| population without clearly defined sampling |  |
| frame or with extensive inclusion/exclusion  criteria |  |
| No method of selection described | 0 |
| **Representativeness of controls** |  |
| Controls were consecutive/randomly drawn | 2 |
| from the same sampling frame |  |
| (ward/community) as cases |  |
| Controls were consecutive/randomly drawn | 1 |
| from a different sampling frame as cases |  |
| Not described | 0 |
| **Ascertainment of asthma** |  |
| Clearly described objective criteria for | 2 |
| diagnosis of asthma |  |
| Diagnosis of asthma by patient self-report or | 1 |
| by patient history |  |
| Not described | 0 |
| **Ascertainment of controls** |  |
| Controls were tested to screen out asthma | 2 |
| Controls were subjects who did not report | 1 |
| asthma |  |
| Not described | 0 |
| **Genotyping examination** |  |
| Genotyping done under blinded condition | 1 |
| Unblinded or not mentioned | 0 |
| **Hardy-Weinberg equilibrium** |  |
| Hardy-Weinberg equilibrium in control group | 2 |
| Hardy-Weinberg disequilibrium in control | 1 |
| group |  |
| No checking for Hardy-Weinberg equilibrium | 0 |
| **Association assessment** |  |
| Assess association between genotypes and | 2 |
| asthma with appropriate statistics and |  |
| adjustment for confounders |  |
| Assess association between genotypes and | 1 |
| asthma with appropriate statistics without |  |
| adjustment for confounders |  |
| Inappropriate statistics used | 0 |
| **Response rate** |  |
| Response rates for both groups are the same | 2 |
| i.e., to within 5% |  |
| Response rates are different, between 5% and | 1 |
| 10% |  |
| Response rates are more than 10% different, | 0 |
| or no mention of response rates |  |
